# Diagnostic value of spectral reconstructions in detecting incidental skeletal muscle metastases in CT staging examinations

**DOI:** 10.1186/s40644-019-0235-3

**Published:** 2019-07-17

**Authors:** Simon Lennartz, Nils Große Hokamp, Nuran Abdullayev, Markus Le Blanc, Andra-Iza Iuga, Grischa Bratke, David Zopfs, David Maintz, Jan Borggrefe, Thorsten Persigehl

**Affiliations:** 1 Department of Diagnostic and Interventional Radiology, University of Cologne, Faculty of Medicine and University Hospital Cologne, Kerpener Str. 62, 50937 Cologne, Germany; 2 Department of Radiology, Case Western Reserve University and University Hospitals, Cleveland, OH USA; 3 Else Kröner Forschungskolleg Clonal Evolution in Cancer, University Hospital Cologne, Weyertal 115b, 50931 Cologne, Germany

**Keywords:** Oncologic imaging, Dual energy computed tomography, Skeletal muscle metastases, Iodine quantification, Iodine maps

## Abstract

**Background:**

To investigate if iodine density overlay maps (IDO) and virtual monoenergetic images at 40 keV (VMI_40keV_) acquired from spectral detector computed tomography (SDCT) can improve detection of incidental skeletal muscle metastases in whole-body CT staging examinations compared to conventional images.

**Methods:**

In total, 40 consecutive cancer patients who underwent clinically-indicated, contrast-enhanced, oncologic staging SDCT were included at this retrospective study: 16 patients with *n* = 108 skeletal muscle metastases confirmed by prior or follow-up CT, ^18^F-FDG-PET, MRI or histopathology, and a control group of 24 patients without metastases. Four independent readers performed blinded, randomized visual detection of skeletal muscle metastases in conventional images, IDO and VMI_40keV_, indicating diagnostic certainty for each lesion on a 5-point Likert scale. Quantitatively, ROI-based measurements of attenuation (HU) in conventional images and VMI_40keV_ and iodine concentration in IDO were conducted. CNR was calculated and receiver operating characteristics (ROC) analysis of quantitative parameters was performed.

**Results:**

Regarding subjective assessment, IDO (63.2 (58.5–67.8) %) and VMI_40keV_ (54.4 (49.6–59.2) %) showed an increased sensitivity for skeletal muscle metastases compared to conventional images (39.8 (35.2–44.6) %). Specificity was comparable in VMI_40keV_ (69.8 (63.2–75.8) %) and conventional images (69.2 (60.6–76.9) %), while in IDO, it was moderately increased to 74.2 (65.3–78.4) %. Quantitative image analysis revealed that CNR of skeletal muscle metastases to circumjacent muscle was more than doubled in VMI_40keV_ (25.8 ± 11.1) compared to conventional images (10.0 ± 5.3, *p* ≤ 0.001). Iodine concentration obtained from IDO and HU acquired from VMI_40kev_ (AUC = 0.98 each) were superior to HU attenuation in conventional images (AUC = 0.94) regarding differentiation between healthy and metastatic muscular tissue (*p* ≤ 0.05).

**Conclusions:**

IDO and VMI_40keV_ provided by SDCT improve diagnostic accuracy in the assessment of incidental skeletal muscle metastases compared to conventional CT.

## Background

Soft-tissue metastases of the skeletal muscle (SMM) are less frequent in comparison to liver, lung, bone, or brain metastases [1–8] indicating a high resistance of muscular tissue to metastatic infestation [9]. However, some malignancies, such as melanoma, gastrointestinal, renal and lung cancer, are associated with a relatively high prevalence of SMM [10]. Detection of these lesions is of high clinical interest, as manifestation of SMM is associated with a worsened disease outcome [11] and might switch the treatment to a palliative approach. Although contrast-enhanced CT is the method of choice for full-body scans in oncologic imaging, it commonly underestimates the prevalence of those particular metastases compared to full-body MRI or PET-CT [12, 13]. It has been shown recently that iodine density overlay maps (IDO) from dual-energy computed tomography (DECT) have the potential for an improved detection of different metastatic conditions including SMM in melanoma patients [14]. In DECT, one high- and one low-energy dataset is acquired from the polyenergetic x-ray spectrum. This can be achieved through different technical approaches: tube-based DECT scanner work with 1) two sources at different tube voltage (dual-source), 2) two sequent rotations at different tube potentials (dual spin), 3) a technique, in which a beam filter is used to split up an x-ray emitted by a single source in two different-energetic partial beams (twin beam) or 4) rapid switching of tube potentials (kVp switching) [15]. In contrast to these source-based concepts, the spectral detector CT (SDCT, Philips Healthcare, Netherlands) operates based on a single-source system working with a dual-layer-detector which registers lower-energetic photons within the upper yttrium-based layer while higher-energetic photons are detected within the lower gadolinium-oxysulphide layer [16]. That approach features concise spatial and angular matching, holding the potential to significantly reduce image noise. Low noise is of special relevance for low keV virtual monoenergetic images (VMI) which have been shown to improve lesion detection of different metastatic conditions, such as pulmonary or hepatic metastasis but are impaired by an increase in image noise at source-based DECT [14, 17, 18]. Another inherent gain of the detector-based approach is that post-processing takes place within the raw data domain while tube-based DECT either requires previous angular/temporal interpolation or only enables post-processing within the projection domain.

The purpose of this study is to investigate whether VMI at 40 keV and IDO obtained with SDCT can improve both detection rate and diagnostic certainty in the assessment of incidental SMM in patients suffering from various malignant primary diseases.

## Methods

### Study cohort

For this retrospective study, institutional review board approval was obtained. A total of 24 patients with known metastatic disease of the skeletal muscle who received clinically indicated, oncologic SDCT staging examinations in the period from June 2016 to July 2017 were identified. For study inclusion, confirmation either by PET-CT/MRI or previous/follow-up CT with progression in size or change during treatment was mandatory (see below: “lesion annotation”). Out of those 24 initially identified patients, 8 patients had to be excluded because of a) unenhanced CT scan due to known allergic reaction to iodinated contrast agent (1 patient), b) hip/spine-implants causing severe metal artifacts that hampered diagnostic assessment of the circumjacent muscle (4 patients), or c) SMM with a long axis diameter (LAD) > 5 cm (3 patients). The remaining 16 patients were included in the SMM group (group A). As negative controls, 24 patients without SMM who received contrast-enhanced SDCT staging examinations in the same date range as group A were included as a control group (group B). Due the known correlation of extent of metastatic disease and prevalence of SMM, patients in group B were chosen to match cancer stages as of group A in order to minimize confounding effects in the blinded, randomized subjective assessment.

### Image acquisition and post-processing

All patients were scanned on a dual layer detector DECT system (IQon, Philips Healthcare, Best, The Netherlands). The clinical scans were performed in cranio-caudal direction while patients remained in a supine position during inspirational breath-hold.

**Fig. 1:**
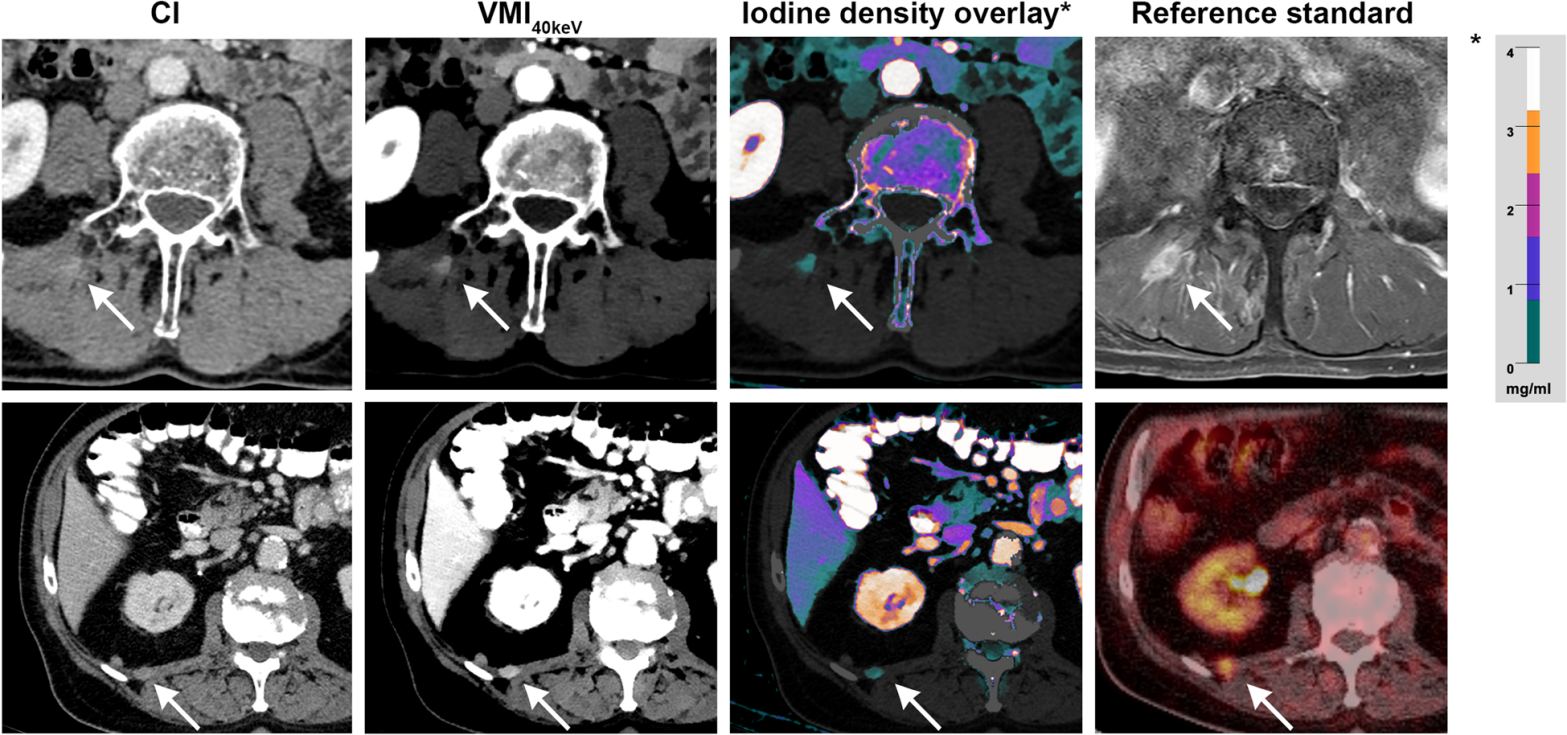
Exemplary cases of skeletal muscle metastases in CI, VMI_40keV_ and IDO with correlating MRI/PET-CT. Upper row: Metastasis of the right autochthone back muscle in a patient with malignant melanoma. Lower row: Patient with lung cancer diagnosed with a metastasis of the right quadratus lumborum muscle. Asterix indicates the color scale used in IDO

Our institution’s monophasic protocol for CT staging examinations contains a portal venous scan of the chest and abdomen triggered by bolus-tracking technique (threshold of 100 HU in the abdominal aorta) with a delay of 70 s. Administration of 120 ml non-ionic, iodinated contrast media bolus (Accupaque 350 mg/ml, GE Healthcare; Little Chalfort, UK) followed by 30 ml saline chaser is routinely performed with an automated injection system at a flow rate of 3.5 ml/s (MEDRAD® Stellant®, Bayer AG, Leverkusen, Germany). The following, constant scan parameters were applied: collimation - 2 × 64 × 0.625 mm; rotation time - 0.5 s; pitch - 0.671; tube current - 120 kVp, matrix - 512 × 512; dose modulation was enabled in all patients (DoseRight 3D-DOM, Philips Healthcare, Best, The Netherlands). Mean CTDIvol was 9.0 mGy. All images were reconstructed with a slice thickness of 2 mm and a 1 mm section increment. Reconstruction of conventional images (CI), VMI and IDO as well as quantitative and qualitative image analysis were performed on a dedicated vendor console (IntelliSpace Portal 9.0, Philips Healthcare, Best, The Netherlands). All spectral results were reconstructed with a dedicated, hybrid iterative spectral reconstruction algorithm (Spectral, Kernel B, level 3). As in clinical routine, conventional images were reconstructed using iDose 4 (level 3). To generate IDO, iodine maps were extracted from the spectral data and fused with CI at 120 kV. Iodine maps are material-specific images calculated from the two different-energetic datasets in which the voxel values represent the iodine concentration of the displayed tissue in mg/ml. Voxels without detectable iodine are equalized to 0 mg/ml and appear black, accordingly. When being fused with the regular greyscale CI, the iodine concentration in IDO appears color-coded as indicated in Fig. 1.

### Lesion annotation

After study inclusion, SDCT scans of the 16 patients with known metastatic disease of the skeletal muscle were evaluated by two radiologists experienced in oncologic imaging who were not involved in the subsequent subjective image analysis. In a consensus, all metastatic lesions of the muscle were identified, and their slice position was recorded. For that, CI, VMI at 40 keV (VMI_40keV_) and iodine density overlay maps (IDO) were used and any suspicious lesion was correlated with the aforementioned reference standard (PET-CT/MRI) and/or previous/follow-up CT scans to confirm lesion dignity reliably. In detail, SMM were defined by the following criteria:

- PET-CT-correlation with clearly enhanced nuclide uptake which was classified malignant by a specialist in nuclear medicine *and/or*
- MRI-correlation with typical morphology *and/or*
- Progression in size without treatment *and/or*
- Progression/regression in size during treatment

### Subjective analysis

To determine diagnostic accuracy and certainty in the assessment of SMM, four blinded radiologists with 4–6 years of experience in oncologic imaging independently reviewed CI, VMI_40keV_ and IDO. For that purpose, the series were randomized and split up to three separate sessions of 40 series each. It was ensured that the same patient did not appear twice within the same session. A latency period of at least four weeks was kept before proceeding with the subsequent session. Within the sessions, the readers were asked to report each lesion suspicious for SMM and name the exact slice position. Additionally, diagnostic certainty regarding lesion dignity was indicated using the following 5-point Likert scale: 1 = most certainly not a metastasis, 2 = likely not a metastasis, 3 = probably a metastasis, 4 = most likely a metastasis, 5 = definitely a metastasis. Standard window settings of IDO were W/L 3/5 for the iodine density map and W/L 60/360 for CI. To maintain a comparable visual perception in of CI and VMI_40keV_, W/L were chosen as 690/240 for the latter; however the readers were free to adjust window settings freely as it is well-known that appropriate window settings are crucial for spectral reconstructions [19, 20]. No time limit for diagnostic assessment was determined.

### Quantitative image analysis

For quantitative image analysis, circular regions of interest (ROIs) were placed within the SMM lesions so that the largest possible area was covered at the slice of the maximum lesion diameter. ROIs within the skeletal muscle and subcutaneous fat were chosen to measure 50 ± 10 mm^2^. After initial placement in CI, ROIs were copied and pasted to VMI and IDO to ensure consistent size and localization. The standard deviation within homogeneous fatty tissue was considered to be representative for image noise.

### Statistical methods

Non-parametric Steel-Dwass test (all pairs) was performed to compare quantitative values (HU, IC, CNR) and subjective Likert scores. Sensitivity, specificity, negative, and positive predictive value (NPV/PPV) of the visual assessment were calculated using a contingency table. Inter-rater agreement was determined using Cohen’s Kappa which was interpreted as suggested in earlier studies: excellent agreement (ĸ ≥ 0.8), good agreement (ĸ ≥ 0.6), moderate agreement (ĸ ≥ 0.4) and poor agreement (ĸ ≤ 0.4) [21, 22]. Receiver operating characteristics (ROC) analysis of HU (CI/VMI_40keV_) and IC was conducted, and Youden’s J was calculated to determine optimal cut-off values. Delong method was used to compare different ROC curves [23]. Statistical significance was determined as *p* ≤ 0.05. Continuous variables are reported as mean ± standard deviation (SD) and Likert scores as median and range. Sensitivities and specificities are given with referring 95% confidence intervals. For statistical analysis, Microsoft Excel (Microsoft Corporation, Redmond, USA), JMP 11 Software (SAS, Cary, USA) and MedCalc 18.2 (Medcalc Software, Ostend, Belgium) were used.

## Results

### Study cohort

SMM group consisted of 16 patients (6 women, 10 men, mean age: 64 ± 12.9 years), control group comprised 24 patients (9 women, 15 men, mean age: 66 ± 12.9 years), showing similar age distribution (*p* = 0.87). Underlying diseases (including melanoma, esophageal cancer, renal cancer, lung cancer, breast cancer, neuroendocrine tumor, and sarcoma) are shown in Table 1.

**Table 1:** Distribution of underlying diseases in patients with skeletal muscle metastases (SMM) and control group patients

Underlying disease	SMM group	Control group
Melanoma	7	3
Esophageal carcinoma	3	4
Pancreatic cancer	0	5
Breast cancer	1	3
Lung cancer	1	1
Renal cell carcinoma	2	0
Sarcoma	1	2
Neuroendocrine tumor	1	2
Gastrointestinal stroma tumor	0	1
Colorectal cancer	0	1
Cholangiocellular carcinoma	0	1
Urothelial carcinoma	0	1

### Subjective analysis

**Fig. 2:**
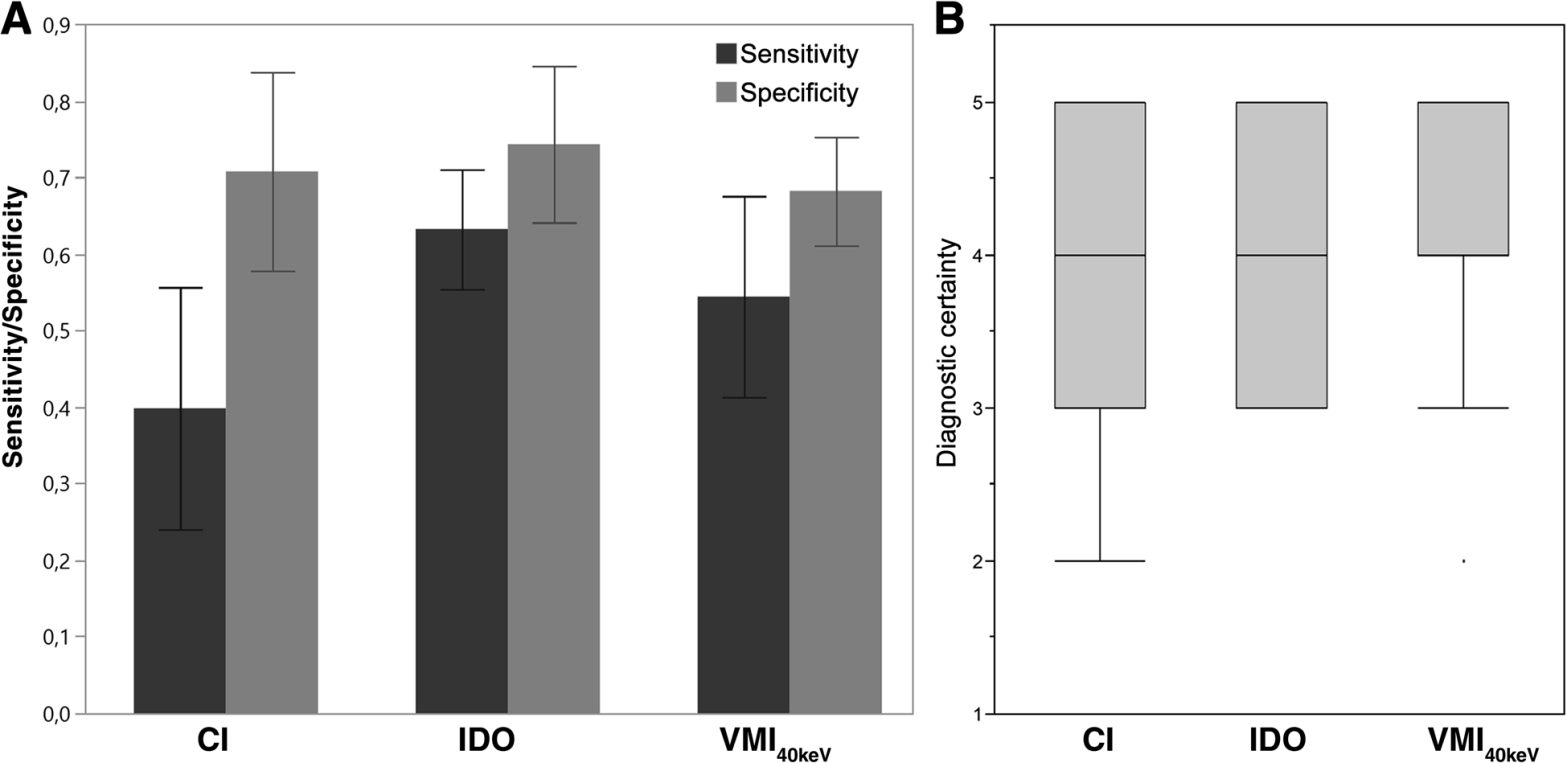
Subjective assessment of skeletal muscle metastases. **a** highlights increased sensitivity for skeletal muscle metastases (SMM) in iodine overlay images (IDO) and virtual monoenergetic images (VMI_40keV_) compared to conventional images (CI). **b** shows median Likert scores for diagnostic certainty which tended to be higher in IDO and VMI_40keV_ but was not statistically significant (p-range:0.16–0.8)

Subjective analysis revealed a higher sensitivity for SMM in IDO (63.2 (58.5–67.8) %) as well as in VMI_40keV_ (54.4 (49.6–59.2) %) compared to CI (39.8 (35.2–44.6) %). Overall specificity was marginally higher in IDO (74.2 (65.3–78.4) %) compared to 69.2 (60.6–76.9) % in CI and 69.8 (63.2–75.8) % in VMI_40keV_. Although indicating a tendency towards better detection of hypervascularized SMM lesions, the influence of iodine concentration on sensitivity was limited: in IDO, sensitivity for lesions with an iodine uptake within the upper two quartiles [1.69–4.32 mg/ml] was 65.5 (58.8–71.7) %, while for the lower two quartiles [0.0–1.69 mg/ml], it was 61.1 (54.3–67.7) %. Diagnostic certainty for correctly identified SMM tended to be superior in VMI_40keV_ (median 5) compared to CI and IDO (median 4) which was not statistically significant (p-range: 0.16–0.8) (Fig. 2). Inter-rater agreement in identifying patients with SMM was good in CI (κ = 0.62) and IDO (κ = 0.76) and moderate in VMI_40keV_ (κ = 0.44). Detailed results of the subjective assessment for every individual reader are shown in Table 2.

**Table 2:** Diagnostic accuracy in the detection of skeletal muscle metastases in conventional images (CI), iodine overlay images (IDO) and virtual monoenergetic images at 40 keV (VMI_40keV_)

READER	Sensitivity/Specificity (%)		
	CI	IDO	VMI_40keV_
1	35.2 (26.2–45.0) / 75.0 (53.3–90.2)	56.5 (46.6–66) / 84.2 (60.4–96.6)	48.1 (38.4–58.0) / 63.6 (40.7–82.8)
2	57.4 (47.5–66.9) / 59.1 (43.4–73.7)	73.1 (63.8–81.2) / 78.8 (67.0–87.9)	64.8 / (55.0–73.8) 74.7 (65.0–82.9)
3	46.3 (36.7–56.2) / 61.8 (43.6–77.8)	65.7 (56.0–74.6) / 60.3 (47.2–72.4)	65.7 (56.0–74.6) / 60.7 (46.8–73.5)
4	20.4 (13.2–29.2) / 87.1 (70.2–96.4)	57.4 (47.5–66.9) / 73.9 (58.9–85.7)	38.9 (29.7–48.8) / 73.7 (56.9–86.6)
OVERALL	39.8 (35.2–44.6) / 69.2 (60.6–76.9)	63.2 (58.5–67.8) / 74.2 (65.3–78.4)	54.4 (49.6–59.2) / 69.8 (63.2–75.8)
Cohen’s Kappa	κ = 0.62	κ = 0.76	κ = 0.44

### Quantitative analysis

**Fig. 3:**
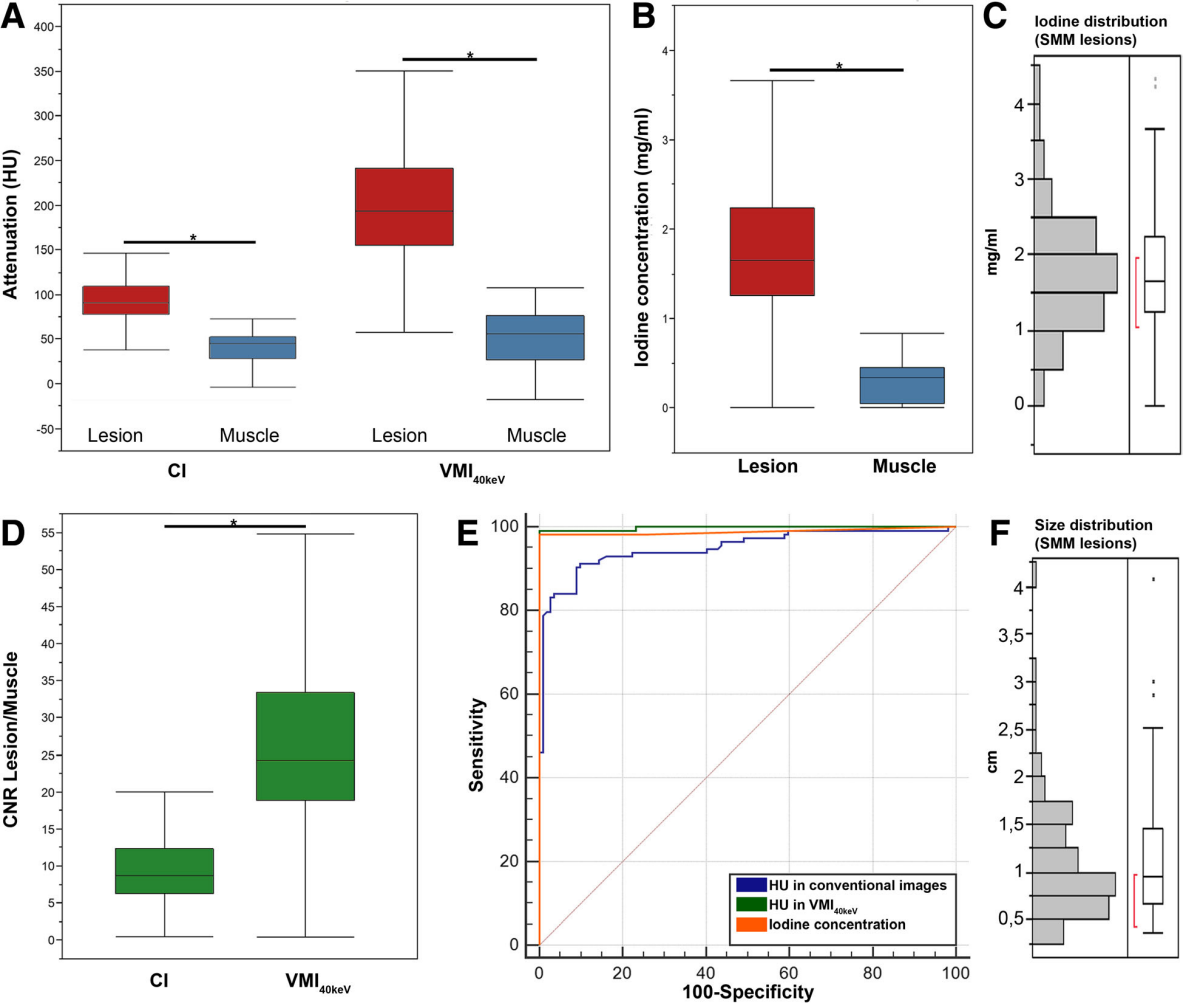
Quantitative analysis of skeletal muscle metastases (SMM). **a** Attenuation of SMM lesions and circumjacent muscle in conventional images (CI) and virtual monoenergetic images (VMI_40keV_). Attenuation of SMM is significantly increased in VMI_40keV_ compared to CI. **b** Iodine concentration [mg/ml] of SMM lesions is significantly higher than in the circumjacent muscle. **c** Distribution of SMM iodine concentration. **d** CNR of SMM and circumjacent muscle is significantly increased in VMI_40keV_ compared to CI. **e** Comparative ROC analysis for discrimination of SMM to circumjacent healthy muscle for attenuation in CI and VMI_40keV_ as well as iodine concentration derived from iodine overlay images (IDO). **f** Distribution of SMM lesion size

Attenuation in CI (lesion: 93.4 ± 26.7, muscle: 45.4 ± 23.3) and VMI_40keV_ (lesion: 194.5 ± 67.9, muscle:50.6 ± 32.7) as well as iodine concentration (lesion: 1.8 ± 0.8 mg/ml, muscle: 0.3 ± 0.2 mg/ml) was significantly higher in SMM compared to circumjacent skeletal muscle (*p* ≤ 0.001). CNR of SMM to circumjacent muscle was significantly increased compared to CI (10.1 ± 5.3) in VMI ranging from 40 to 60 keV (*p* ≤ 0.001) with a maximum CNR of 25.8 ± 11.1 in VMI_40keV_ (*p* ≤ 0.001)_._ VMI at 70 keV yielded an CNR equivalent to CI (10.5 ± 5.3; *p* = 0.3), while in VMI from 80 to 200 keV CNR was significantly lower (*p* ≤ 0.05). ROC analysis yielded a superior differentiation of SMM and healthy muscle in VMI_40keV_ and IDO (AUC = 0.98 each) compared to CI (AUC = 0.94, *p* ≤ 0.05). Youden’s indices revealed optimal cut offs of > 62.1 HU for CI, > 107.7 HU for VMI_40keV_ and > 0.73 mg/ml for IDO. 4 out of 108 included lesions (3.7%) showed necrosis with corresponding decrease in mean iodine uptake to 0.5 ± 0.4 mg/ml compared to 1.8 ± 0.8 mg/ml in non-necrotic lesions (*p* ≤ 0.05; Fig. 3 f). Mean long axis diameter of SMM lesions was 1.1 ± 0.6 cm. Distribution of lesion size is shown in Fig. 3f.

## Discussion

This study investigates if and to what extent iodine density overlay maps (IDO) and virtual monoenergetic images obtained with spectral detector computed tomography (SDCT) can improve visual and quantitative diagnostic accuracy in assessing incidental skeletal muscle metastases (SMM). In CT, the assessment of the muscular system regarding metastatic lesions is severely hampered by different factors: the skeletal muscles constitute a large volume to investigate and usually show high attenuation so that iso- and moderately hyperattenuating metastases may get obscured. In the past, dual energy CT (DECT)-derived reconstructions highlighting the iodine signal have been shown to increase contrast and improve depiction of malignant lesions such as liver metastases, hepatocellular carcinoma, pancreatic or lung tumors [24–29]. This can be achieved using iodine-specific maps or lower-energetic virtual monochromatic images which result in a higher x-ray absorption of iodine as the energy level is moved closer to its absorption maximum, the k-edge (33.2 keV) [30].

Our study revealed that VMI at 40 keV (VMI_40keV_) allowed for a significantly improved contrast of SMM in comparison to the circumjacent tissue resulting in a moderately increased detection rate of those lesions. These results are in line with earlier studies that described improved detection of hyperattenuating lesions in low-energy virtual monoenergetic images [26]. However, in iodine density overlay maps (IDO) in which the iodine signal is color-coded and merged with conventional images (CI), the observed boost in sensitivity compared to CI was markedly higher than in VMI_40keV_. On the other hand, specificity was only slightly higher in IDO and comparable between VMI_40keV_ and CI. A recent study by Uhrig et al. investigated subjective assessment of different distant metastases in patients with melanoma using iodine-overlay images derived from a dual-source CT scanner including a minor subgroup of 13 SMM lesions [14]. They reported a sensitivity for SMM of 8% in conventional images which was boosted up to 99% in iodine overlay images. Compared to these results, overall sensitivity in CI observed in our study was much higher (39.8 (35.2–44.6) %) while in IDO it was lower (63.2 (58.5–67.8) %) which might be explicable by the very small sample size of the SMM subgroup Uhrig et al. examined. In our study, inter-rater agreement for the subjective assessment of SMM was good in CI (κ = 0.62) and IDO (κ = 0.76) and moderate in VMI_40keV_ (κ = 0.44). The observed decrease in inter-rater agreement and specificity in VMI_40keV_ might be owed to the fact that iodine contrast boost may have led to misinterpretation of inhomogeneous muscular contrast enhancement in some cases resulting in false positives. The subjective results are in line with the quantitative image analysis which revealed HU acquired from VMI_40keV_ and iodine concentration obtained from IDO to be superior to conventional HU in differentiating metastatic lesions from circumjacent, unaffected muscle.

Given the results of the subjective assessment, IDO seem the most predestined spectral reconstruction to screen for incidental metastatic muscle infiltration in regular, clinically-indicated CT staging examinations of the chest, abdomen and pelvis. Consequently, they have been implemented into the clinical workflow at our institute for patients suffering from diseases with increased pre-test probability for SMM: melanoma, gastrointestinal tumors, renal cancer, lung cancer, thyroid gland carcinoma, breast cancer, and carcinoma of unknown primary (CUP) [10]. Still, it needs to be stated that the sensitivity of 63% yielded by IDO, although being superior to conventional CT images, is still suboptimal for oncologic imaging purposes. Thus, for some indications, such as staging of metastatic melanoma, ^18^F-FDG PET/CT reflects the best imaging method. However, in staging examinations for patients who do not qualify for regular oncologic follow-up with ^18^F-FDG PET/CT, such as patients with metastatic renal cancer, IDO may help to detect incidental SMM.

Aside from the retrospective nature of our study, there are various limitations: First, the number of 16 included patients with SMM is relatively small. However, considering the number of 108 included lesion and the blinded, randomized study design with 4 independent subjective readers, significance of results still seems sufficiently powered. Overall, the number of eligible patients for inclusion was limited due to the overall low prevalence of metastatic disease of the skeletal muscle as well as the required reference standard necessary for study inclusion. Due to the small patient number there is the possibility of recognition bias which may have influenced the diagnostic accuracy. Another factor that may play a role in this context is possible localization of lesions at memorable sites such as extremities. Recognition effects were minimized as far as possible by a large time frame of at least 4 weeks and randomization of patient/series between repetitive subjective examinations. Still, blinding to reconstructed series was not possible as CI, VMI_40keV_ and IDO can be easily recognized due to their characteristic visual impression. Secondly, it is known that individual intravenous iodine load varies between different scans dependent on circulation and other physiological and technical factors which might potentially influence the iodine density of SMM lesions. Nonetheless, unlike previous studies on DECT-derived iodine concentration, normalization to intravenous iodine load was waived in our study as SMM were allocated within the complete thoracoabdominal scan volume so that normalization to a central vessel e.g. abdominal aorta did not seem to be representative. Lastly, the majority of included SMM lesions were iso- or hyperattenuating in relation to circumjacent skeletal muscle; in fact, only 4 out of 108 included lesions showed central necrosis. This relatively small proportion of necrotic lesions might be on account of the initial exclusion of lesions with a long axis diameter > 5 cm. As large lesions tend to show necrosis more frequently, the study cohort might be biased in terms of these necrotic lesions. However, large SMM might be also easily depicted without the use of spectral reconstructions. As a limitation due to the relatively small sample size, it was not possible to statistically determine if there was a correlation between lesion necrosis and detection rate.

## Conclusions

Iodine overlay images obtained with spectral detector CT improve visual and quantitative diagnostic accuracy in assessing skeletal muscle metastases (SMM) compared to conventional images. Hence, these reconstructions seem advantageous at regular oncological CT examinations regarding the detection of incidental SMM particularly for patients suffering from metastatic malignancies and yet not qualifying for routine ^18^F-FDG PET/CT imaging.

## Data Availability

Please contact the corresponding author for data requests.

## Abbreviations

CI: Conventional images
DECT: Dual-energy computed tomography
IC: Iodine concentration
IDO: Iodine density overlay maps
SDCT: Spectral detector computed tomography
SMM: Skeletal muscle metastases
VMI_40keV_: Virtual monoenergetic images at 40 keV
